# Association between homologous recombination deficiency status and carboplatin treatment response in early triple-negative breast cancer

**DOI:** 10.1007/s10549-024-07436-1

**Published:** 2024-07-24

**Authors:** Zheng Wang, Yujie Lu, Mengyuan Han, Anqi Li, Miao Ruan, Yiwei Tong, Cuiyan Yang, Xiaotian Zhang, Changbin Zhu, Chaofu Wang, Kunwei Shen, Lei Dong, Xiaosong Chen

**Affiliations:** 1 Department of General Surgery, Comprehensive Breast Health Center, Ruijin Hospital, Shanghai Jiao Tong University School of Medicine, 197 Ruijin Er Road, Shanghai, 200025 China; 2 Department of Pathology, Ruijin Hospital, Shanghai Jiao Tong University School of Medicine, 197 Ruijin Er Road, Shanghai, 200025 China; 3 Department of Translational Oncology, Amoy Diagnostics Co., Ltd., Xiamen, 361026 China

**Keywords:** Triple-negative breast cancer, Homologous recombination deficiency, BRCA, Pathological response, Prognosis

## Abstract

**Background:**

The aim of this study was to assess homologous recombination deficiency (HRD) status and its correlation with carboplatin treatment response in early triple-negative breast cancer (TNBC) patients.

**Methods:**

Tumor tissues from 225 consecutive TNBC patients were evaluated with an HRD panel and homologous recombination-related (HRR) gene expression data. HRD positivity was defined as a high HRD score and/or *BRCA1/2* pathogenic or likely pathogenic mutation. Clinicopathological factors, neoadjuvant treatment response, and prognosis were analyzed with respect to HRD status in these TNBC patients.

**Results:**

HRD positivity was found in 53.3% of patients and was significantly related to high Ki67 levels (*P* = 0.001). In patients who received neoadjuvant chemotherapy, HRD positivity (*P* = 0.005) or a high HRD score (*P* = 0.003) was significantly associated with a greater pathological complete response (pCR) rate, especially in those treated with carboplatin-containing neoadjuvant regimens (HRD positivity vs. negativity: 50.00% vs. 17.65%, *P* = 0.040). HRD positivity was associated with favorable distant metastasis-free survival (hazard ratio HR 0.49, 95% confidence interval CI 0.26–0.90, *P* = 0.022) and overall survival (HR 0.45, 95% CI 0.20–0.99, *P* = 0.049), irrespective of carboplatin treatment.

**Conclusion:**

TNBC patients with high HRDs had high Ki67 levels and *BRCA* mutations. HRD-positive TNBC patients treated with carboplatin had a higher pCR rate. Patients with HRD positivity had a better prognosis, irrespective of carboplatin treatment, warranting further evaluation.

**Supplementary Information:**

The online version contains supplementary material available at 10.1007/s10549-024-07436-1.

## Introduction

Breast cancer is the most common malignancy worldwide [1]. Increasing efforts have been made in searching for tools to guide physicians in making precise therapeutic choices and in predicting the prognosis of the disease. Among these new tools, homologous recombination deficiency (HRD), which is known to be a vital motif in the pathogenesis, progression, and treatment efficacy of breast cancer, could be of interest [2]. Mutations in *BRCA1* and *BRCA2* are the most well-known causes of HRD, and HRD can occur secondary to germline or somatic alterations of other homologous recombination (HR)-related genes or *BRCA1* promoter methylation [3].

Current data revealed that HRD positivity occurs in approximately 30% of breast cancer patients, and triple-negative breast cancer (TNBC) has the highest rate of HRD, which is more than 60% according to several small cohort analyses [4, 5]. Through whole-genome sequencing analyses of triple-negative breast cancer (TNBC) patients, Staaf et al. revealed that among TNBC patients with a high HRDetect mutational signature, 67% of cases were caused by germline/somatic *BRCA1/2*, as well as by other genomic/epigenic abnormalities, such as *BRCA1* promoter hypermethylation, *RAD51C* hypermethylation, or biallelic loss of *PALB2*, illustrating the existence of many alternative alterations that may lead to HRD tumor status [6]. In a pancancer study based on TCGA data, Su et al. demonstrated inferior overall survival (OS) in HRD score-high patients compared to HRD score-low patients [7]. However, another study based on the Swedish database revealed better invasive disease-free survival (iDFS) in patients with HRD-high tumors than in those with HRD-low tumors [8]. Therefore, the prognostic value of HRD status in TNBC patients remains unclear.

In terms of treatment approaches, homologous recombination repair (HRR) is an important DNA repair pathway for DNA damage and mostly involves DNA double strands and interstrand cross-links [9]. Thus, tumors with HRD are considered to be more genomically unstable and immunogenic and therefore potentially have a greater number of nonsynonymous mutations, leading to more tumor neoantigens [10–12]. Given the high prevalence of HRD in TNBC and the mechanism of synthetic lethality, the use of additional therapies targeting HRD, such as platinum salts and poly ADP-ribose polymerase (PARP) inhibitors, has greatly improved the treatment response. In neoadjuvant chemotherapy (NAC), adding carboplatin to anthracycline/taxane significantly increased the pathological complete response (pCR) rate in TNBC patients [13, 14]. However, according to several NAC trials, *BRCA* mutations were not correlated with a higher pCR rate in TNBC patients receiving platinum agents [15–17]. In addition to *BRCA* mutation, Telli et al. found that a high HRD is associated with a better response to platinum-containing NAC in a pooled analysis of three neoadjuvant trials [18]. Moreover, exploratory analysis of the BrighTNess and GeparSixto trials demonstrated that HRD status was an independent predictor of the pCR rate but not the carboplatin treatment response [19, 20]. Taken together, the current evidence is insufficient to support routine testing of HRD status to guide the use of platinum salt in daily practice.

In the present study, we aimed to analyze the frequency of HRD positivity and to assess the association between HRD status and carboplatin treatment response in early TNBC patients.

## Methods

### Patients and samples

We retrospectively screened consecutive breast cancer patients treated at the Comprehensive Breast Health Center, Ruijin Hospital, Shanghai Jiao Tong University School of Medicine (RJBC-CBHC) from January 1, 2012 to July 31, 2022. Patients who met the following eligibility criteria were included: (1) had invasive breast cancer, (2) were pathologically diagnosed with TNBC, (3) had available formalin-fixed paraffin-embedded (FFPE) tissues, and (4) were evaluated with HRR genotyping and HRD assays. The exclusion criteria were as follows: (1) male breast cancer, (2) de novo stage IV, and (3) incomplete immunohistochemistry (IHC) information. All tumor size, lymph node status, comorbidities, and adjuvant therapy strategies were permitted.

Archival FFPE blocks were selected from the biobank at the Department of Pathology, Ruijin Hospital. Elaborate clinical data were retrieved from the Shanghai Jiao Tong University Breast Cancer Database (SJTU-BCDB). All patients provided informed consent, and our study was approved by the Ethical Committees of Ruijin Hospital, Shanghai Jiao Tong University School of Medicine. All procedures were in accordance with the 1964 Helsinki Declaration and its later amendments.

### Assessment of clinicopathological information

At least two experienced pathologists (A. Li and M. Ruan) from the Department of Pathology, Ruijin Hospital, Shanghai Jiao Tong University School of Medicine, contributed to the tumor histopathological analysis. IHC was used to determine the status of the estrogen receptor (ER), progesterone receptor (PR), and human epidermal growth factor receptor-2 (HER2) as well as the proliferation index (Ki67). ER and PR positivity were defined as no less than 1% stained nuclei, as described in our previous publications [21, 22]. Ki67 > 30% was classified as high expression. HER2 status was classified as “HER2 low” if the IHC results were HER2 1 + or HER2 2 + /fluorescence in situ hybridization (FISH) negative and HER2 0 patients were defined as “HER2 negative.”

### Evaluation of the efficacy of neoadjuvant chemotherapy

Among the patients included, patients who underwent neoadjuvant chemotherapy composed the neoadjuvant cohort for neoadjuvant chemotherapy efficacy analysis. The efficacy of neoadjuvant chemotherapy was evaluated according to the pathological result of the final surgical resection sample. A pathological complete response was defined as the disappearance of the target lesion(s).

### Follow-up

All patients underwent regular outpatient follow-ups or follow-up calls once every 3 months within the first 2 years after surgery, once every 6 months through the third to the fifth year, and once every year thereafter, in accordance with the American Society of Clinical Oncology Guidelines (ASCO guidelines) [23]. DMFS was defined as the time interval between surgery and the event of distant recurrence, death from breast cancer, death from nonbreast cancer, or death from an unknown cause. OS was defined as the time interval between surgery and death from breast cancer, death from nonbreast cancer or death from an unknown cause [24]. For patients with no events, DMFS and OS were defined as the time interval between surgery and the last follow-up date (June 30, 2020).

### HRD Testing

FFPE tumor tissues were stained with hematoxylin and eosin (H&E) and assessed by two pathologists (A. Li, M. Ruan) to determine tumor purity. For patients received neoadjuvant chemotherapy, pre-treatment samples collected by core needle biopsy were used for HRD test. If the tumor purity was ≥ 20%, the tumor tissue was subjected to DNA extraction. The ReliaPrep FFPE gDNA Miniprep System (Promega) was used to extract genomic DNA from FFPE tumor tissues. A total of 100 ng of DNA was used for library construction and hybrid capture with the AmoyDx® HRD complete panel, which selected coding sequence (CDS) regions for 24,000 single-nucleotide polymorphisms (SNPs) and 20 genes (*ATM*, *BARD1*, *BRCA1*, *BRCA2*, *BRIP1*, *CDH1*, *CDK12, CHEK1*, *CHEK2*, *FANCA*, *FANCL*, *HDAC2*, *PALB2*, *PPP2R2A*, *PTEN*, *RAD51B*, *RAD51C*, *RAD51D*, *RAD54L*, *TP53*). DNA libraries were sequenced on an Illumina NovaSeq 6000 platform with 150 paired-end reads.

The processed raw data were mapped to a reference (human reference genome, hg19) and corrected by UMI for statistical analysis. According to the procedures recommended by the manufacturer [25], a genomic scar (GS) model was built to predict homologous recombination deficiency events via a novel machine learning-based algorithm. A genomic scar score (GSS) ≥ 50.0 was considered to indicate GSS positivity. The gene variations involved in the CDS regions of 20 genes included base substitutions and small insertions and deletions. In addition, 20 gene variants were classified according to the American College of Medical Genetics guidelines [26]. HRD positivity was defined as a GSS ≥ 50.0 and/or *BRCA1/2* pathogenic or likely pathogenic mutation.

### Statistical analysis

The data distribution was characterized by frequency tabulation and summary statistics. Differences in continuous data were assessed through Student’s *t* test or the Wilcoxon rank sum test. Differences in categorical data were assessed through the chi-square test or Fisher’s exact test. Correlations between categorical data were assessed through the Spearman correlation coefficient. *K*‒*M* curves and the log-rank test were used to compare unadjusted survival between study groups. Cox proportional hazards models were used to evaluate hazard ratios across subgroups and to adjust for patients’ clinicopathological and therapeutic parameters. A two-sided *P* < 0.05 was considered to indicate statistical significance. All the graphs were generated using GraphPad Prism version 10.0.0 (GraphPad Software, San Diego). All the statistical analyses were performed using Stata version 16.1 (StataCorp LP, College Station, Texas).

## Results

### Baseline characteristics

**Fig. 1:**
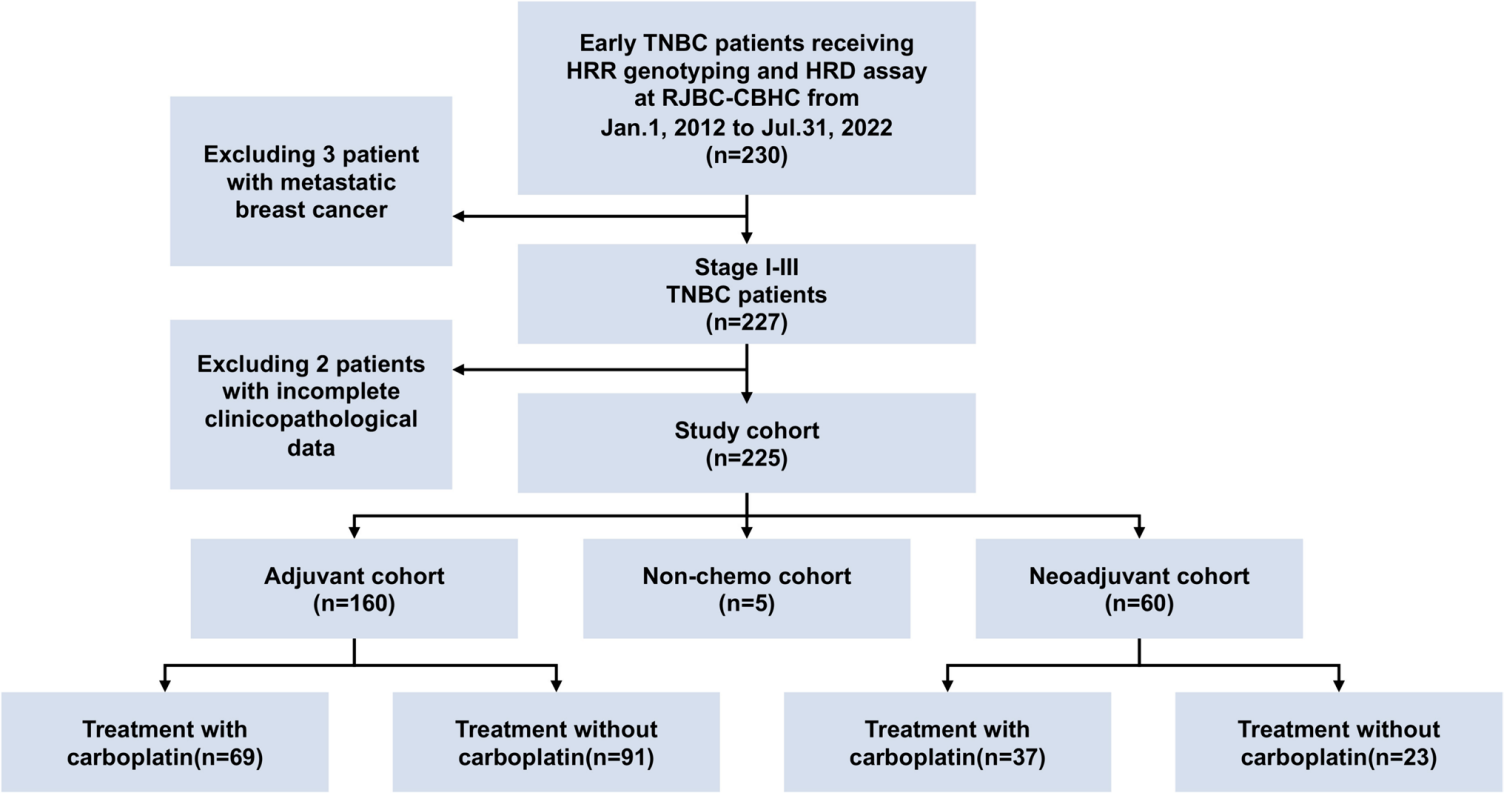
Study flowchart. *TNBC* triple-negative breast cancer, *HRR* homologous recombination repair, *HRD* homologous recombination deficiency, *RJBC-CBHC* Ruijin Hospital Comprehensive Breast Health Center

A total of 225 patients who received radical surgery (Fig. 1), HRR genotyping, and HRD assays were included in the final analysis, of whom 120 (53.3%) were HRD positive (Table 1). Baseline clinical features and pathological characteristics according to HRD status are shown in Table 1. A higher percentage of HRD-positive patients was observed in TNBC patients younger than 35 (16.67% vs 7.62%). HRD positivity was significantly associated with high tumor grade (71.67% vs 51.43%, *P* = 0.001) and high Ki67 levels (83.33% vs 54.29%, *P* < 0.001), indicating more progressive disease. Multivariate analysis revealed that only Ki67 > 30% was independently associated with a high HRD (odds ratio OR 3.21, 95% confidence interval CI 1.66–6.20; *P* = 0.001).

**Table 1:** Clinical pathological characteristics stratified by HRD mutation status

	Total (%)	HRD ( +)	HRD (−)	*P*
Total	225	120	105	
Age				0.058
≤ 35	28 (12.44)	20 (16.67)	8 (7.62)	
36–75	190 (84.44)	98 (81.67)	92 (87.62)	
> 75	7 (3.11)	2 (1.67)	5 (4.76)	
Pathology				0.039
IDC	190 (84.44)	102 (85.00)	88 (83.81)	
ILC	5 (2.22)	2 (1.67)	3 (2.86)	
Apocrine carcinoma	4 (1.78)	0 (0.00)	4 (3.81)	
Medullary carcinoma	2 (0.89)	2 (1.67)	0 (0.00)	
Metaplastic breast carcinoma	12 (5.33)	5 (4.17)	7 (6.67)	
Mucinous adenocarcinoma	1 (0.44)	0 (0.00)	1 (0.95)	
Invasive carcinoma (unidentified)	11 (4.89)	9 (7.50)	2 (1.90)	
Grade				0.002
I	1 (0.44)	1 (0.83)	0 (0.00)	
II	56 (24.89)	19 (15.83)	37 (35.24)	
III	140 (62.22)	86 (71.67)	54 (51.43)	
Unclassified	28 (12.44)	14 (11.67)	14 (13.33)	
Ki67				< 0.001
≤ 30%	68 (30.22)	20 (16.67)	48 (45.71)	
> 30%	157 (69.78)	100 (83.33)	57 (54.29)	
Clinical T Stage				0.766
T1	68 (30.22)	39 (32.50)	29 (27.62)	
T2	140 (62.22)	72 (60.00)	68 (64.76)	
T3	16 (7.11)	8 (6.67)	8 (7.62)	
T4	1 (0.44)	1 (0.83)	0 (0.00)	
Clinical N Stage				0.274
N0	90 (40.00)	43 (35.83)	47 (44.76)	
N1	76 (33.78)	47 (39.17)	29 (27.62)	
N2	37 (16.44)	20 (16.67)	17 (16.19)	
N3	22 (9.78)	10 (8.33)	12 (11.43)	
Breast/ovarian cancer family history				0.924
No	187 (83.11)	100 (83.33)	87 (82.86)	
Yes	38 (16.89)	20 (16.67)	18 (17.14)	
Family history of other cancers				0.161
No	211 (93.78)	110 (91.67)	101 (96.19)	
Yes	14 (6.22)	10 (8.33)	4 (3.81)	
Second primary cancer				0.310
No	216 (96.00)	117 (97.50)	99 (94.29)	
Yes	9 (4.00)	3 (2.50)	6 (5.71)	
Surgery				0.023
BCS	75 (33.33)	48 (40.00)	27 (25.71)	
Mastectomy	150 (66.67)	72 (60.00)	78 (74.29)	
Neoadjuvant chemotherapy				0.365
No	165 (73.33)	91 (75.83)	74 (70.48)	
Yes	60 (26.67)	29 (24.17)	31 (29.52)	
Adjuvant chemotherapy				0.169
No	65 (28.89)	30 (25.00)	35 (33.33)	
Yes	160 (71.11)	90 (75.00)	70 (66.67)	
Treatment with carboplatin				0.353
No	119 (52.89)	60 (50.00)	59 (56.19)	
Yes	106 (47.11)	60 (50.00)	46 (43.81)	
Radiotherapy				0.096
No	63 (28.00)	28 (23.33)	35 (33.33)	
Yes	162 (72.00)	92 (76.67)	70 (66.67)	

*HRD* homologous recombination deficiency, *IDC* invasive ductal carcinoma, *BCS* breast-conserving surgery

### HRD score and non-HRR gene mutation status

**Fig. 2:**
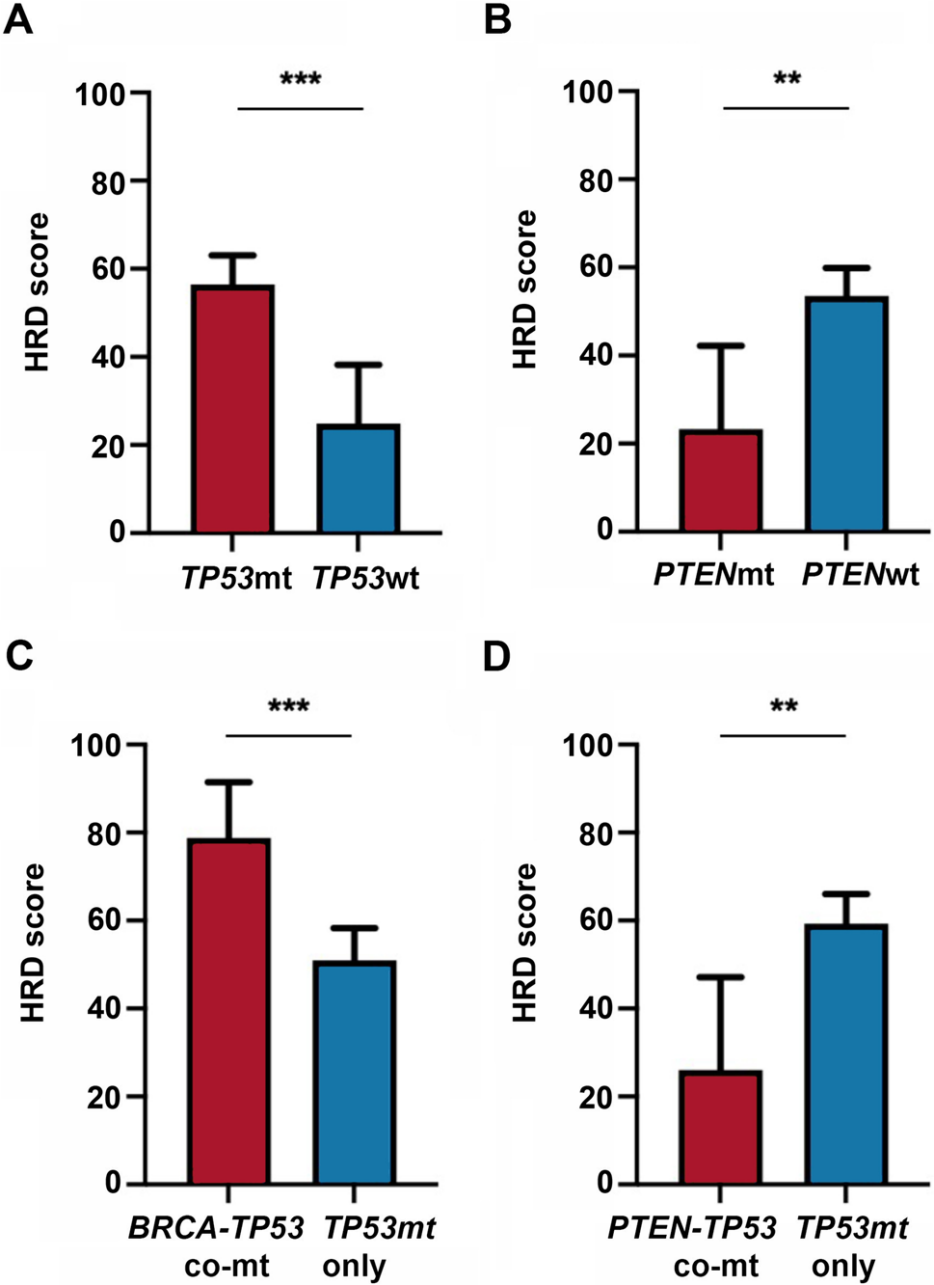
Distribution of HRD scores stratified by non-HRR gene mutation status. **A** Distribution of HRD scores stratified by *TP53* mutation status. **B** Distribution of HRD scores stratified by *PTEN* mutation status. **C** Distribution of HRD scores stratified by *BRCA-TP53* comutation status. **D** Distribution of HRD scores stratified by *PTEN-TP53* comutation status *HRR* homologous recombination repair, *HRD* homologous recombination deficiency *P < 0.05; **P < 0.01; ***P < 0.001; *NS* not significant

Regarding the non-*HRR* gene mutations tested in the current panel, 83.11% (*n* = 187) and 8.00% (*n* = 18) of patients were found to carry *TP53* and *PTEN* mutations, respectively. *TP53* (*P* < 0.001, Fig. 2A) and *PTEN* (*P* < 0.010, Fig. 2B) mutations were significantly related to the HRD score. Compared with wild-type *TP53*, mutated TP53 was related to a greater HRD score (56.48 ± 6.52 vs 24.86 ± 13.37, *P* < 0.001), while *PTEN*-mutated tumors had a lower HRD score than wild-type PTEN (23.17 ± 18.94 vs 53.37 ± 6.28, *P* = 0.008). Specifically, *BRCA-TP53* comutation carriers had significantly greater HRD scores than those who had only *TP53* mutations (78.82 ± 12.63 vs 50.97 ± 7.31, *P* < 0.001; Fig. 2C), while *PTEN-TP53* comutation carriers had significantly lower HRD scores than those who had only *TP53* mutations (26.04 ± 21.08 vs 59.33 ± 6.76, *P* < 0.01; Fig. 2D).

### HRD status and neoadjuvant treatment response

**Fig. 3:**
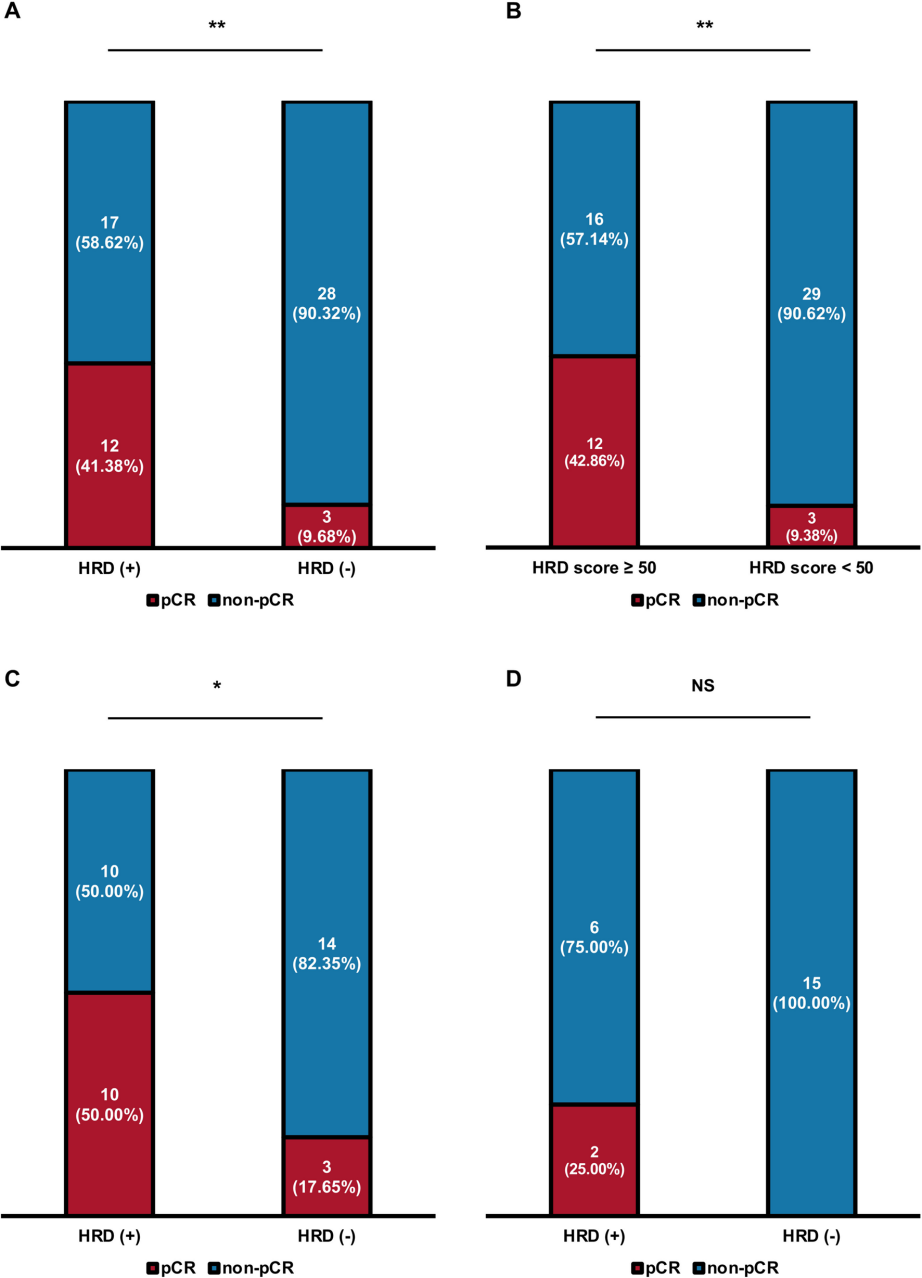
Association between HRD and neoadjuvant chemotherapy efficacy. **A** Distribution of neoadjuvant chemotherapy efficacy stratified by HRD status in all patients (*P* = 0.005). **B** Distribution of neoadjuvant chemotherapy efficacy stratified by HRD score groups in all patients (*P* = 0.003). **C** Distribution of neoadjuvant chemotherapy efficacy stratified by HRD status in patients treated with carboplatin (*P* = 0.040). **D** Distribution of neoadjuvant chemotherapy efficacy stratified by HRD status in patients treated without carboplatin (*P* = 0.111). *HRD* homologous recombination deficiency, *pCR* pathological complete response. *P < 0.05; **P < 0.01; ***P < 0.001; *NS* not significant

Overall, 60 of 225 patients received NAC (Fig. 1) and 15 (25.00%) patients achieved pCR. Both HRD positivity and an HRD score ≥ 50.0 were significantly associated with a higher pCR rate after receiving NAC. The pCR rate was 41.38% in HRD-positive patients and 9.68% in HRD-negative patients (*P* = 0.005, Fig. 3A). Patients with HRDs ≥ 50.0 had higher pCR rates than did those with HRDs ≤ 50.0 (42.86% vs 9.38%, *P* = 0.003; Fig. 3B).

Regarding HRD status and platinum neoadjuvant efficacy, in 37 patients who received carboplatin-containing NAC regimens, HRD positivity was significantly associated with a higher pCR rate (50.00% vs 17.65%, *P* = 0.040; Fig. 3C), while in the remaining 23 patients who were not treated with carboplatin, only a trend toward a greater pCR rate was observed in HRD-positive patients (25.00% vs 0.00%, *P* = 0.111; Fig. 3D).

### HRD status and survival

**Fig. 4:**
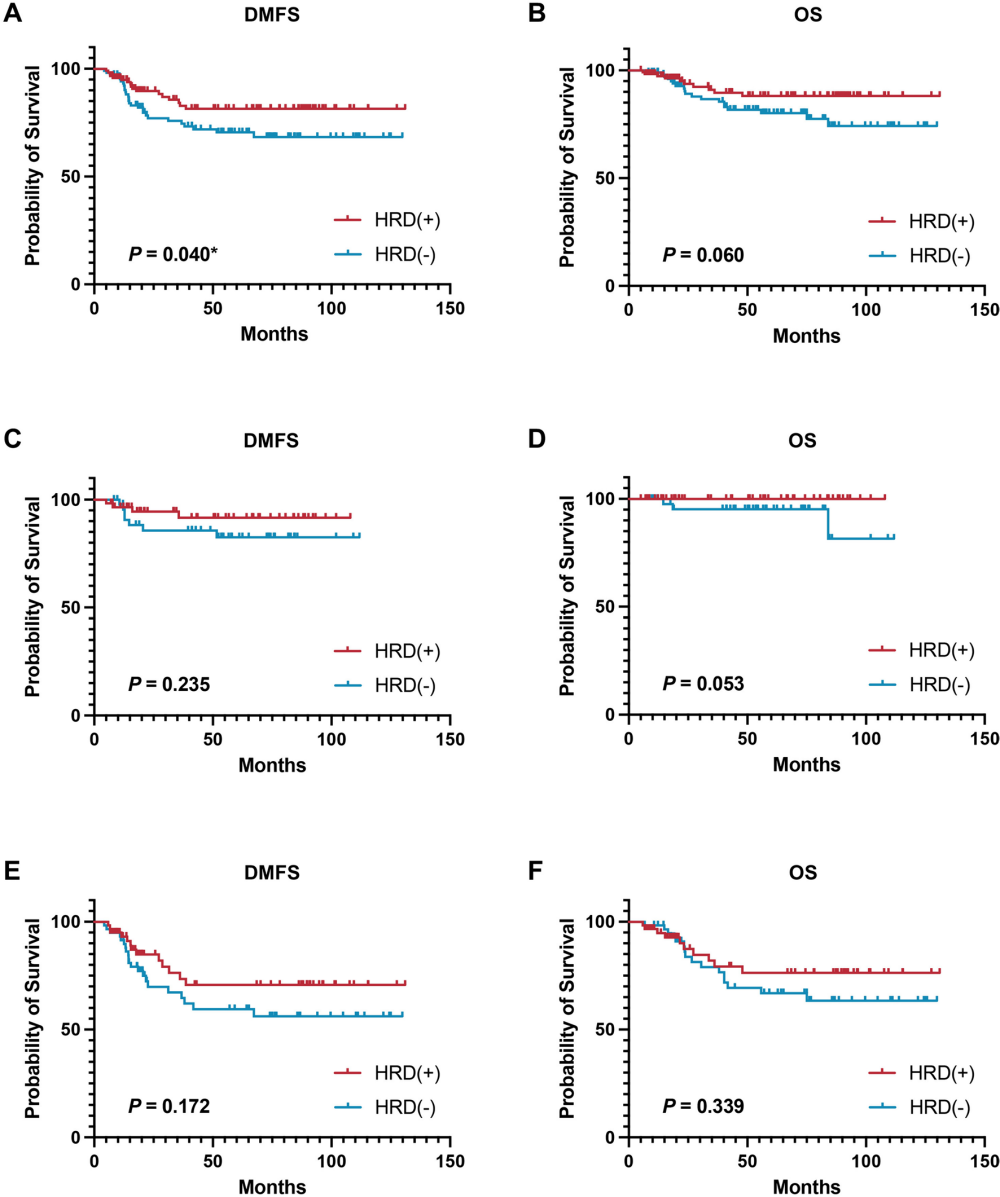
Prognostic significance of HRD status. **A** Distant metastasis-free survival in all patients. **B** Overall survival in all patients. **C** Distant metastasis-free survival in patients treated with carboplatin. **D** Overall survival in patients treated with carboplatin. **E** Distant metastasis-free survival in patients treated without carboplatin. **F** Overall survival in patients treated without carboplatin. *HRD* homologous recombination deficiency, *DMFS* Distant metastasis-free survival, *OS* Overall survival

After a median follow-up of 50.9 months, 29 deaths and 54 disease relapse events occurred in the whole population. As shown by the Kaplan‒Meier curves in Fig. 4, HRD-positive carriers had a significantly better DMFS (*P* = 0.040, Fig. 4A) and a trend toward better OS (*P* = 0.060, Fig. 4B) than HRD-negative carriers. Multivariate analysis revealed that after adjustment for other clinicopathological characteristics including clinical T stage, clinical N stage, and HRD status was independently associated with improved DMFS (hazard ratio HR 0.49, 95% CI 0.26–0.90, *P* = 0.022; Table 2) and OS (HR 0.45, 95% CI 0.20–0.99, *P* = 0.049; Table 3). Considering the presence of carboplatin, HRD positivity was associated with a trend toward superior DMFS (with carboplatin, *P* = 0.235; Fig. 4C**;** without carboplatin, *P* = 0.172; Fig. 4E) and OS (with carboplatin, *P* = 0.053; Fig. 4D**;** without carboplatin, *P* = 0.339; Fig. 4F) in the whole population. There was no significant interaction effect on the prognostic value of HRD status and carboplatin treatment (*P* interaction = 0.730 for DMFS, Fig. 4C; *P* interaction = 1.000 for OS, Fig. 4D). Moreover, HRD status had a similar effect on OS and DMFS in the other subgroups (all *P* values > 0.05, Supplementary Fig. S2). Taking neoadjuvant treatment setting into account, we found that HRD status has consistent significant prognostic value not matter in patients received NAC or underwent upfront surgery (DMFS, *P* for interaction 0.719; OS, *P* for interaction 0.452, Supplementary Fig. S2).

**Table 2:** Univariate and multivariate analyses of distant metastasis-free survival

Clinicopathological parameter	Hazard ratio	95% CI	*P*
Univariate analysis			
Age (≤ 35 versus 36–75 versus > 75)	≤ 35 versus 36–75: 2.18 ≤ 35 versus > 75:3.49	≤ 35 versus 36–75: 0.67–7.07 ≤ 35 versus > 75:0.58–20.89	0.271
Pathology IDC versus others	4.05	0.98–16.74	0.053
Grade III versus I–II	0.68	0.37–1.27	0.226
Ki67 > 30% versus ≤ 30%	1.46	0.74–2.89	0.279
cT2–T4 versus cT1	4.91	1.76–13.73	0.002
cN + versus cN0	3.66	1.63–8.21	0.002
Breast/ovarian cancer family history versus not	0.69	0.29–1.63	0.393
Family history of other cancers versus not	0.90	0.28–2.93	0.868
Second primary cancer versus not	0.51	0.07–3.70	0.505
Chemotherapy versus not	1.22	0.17–8.89	0.843
Radiotherapy versus not	1.40	0.71–2.78	0.332
HRD + versus HRD-	0.53	0.29–0.98	0.044
Multivariate analysis			
cT2–T4 versus cT1	3.71	1.31–10.53	0.014
cN + versus cN0	3.12	1.37–7.09	0.007
HRD + versus HRD−	0.49	0.26–0.90	0.022

*HRD* homologous recombination deficiency, *BCS* breast-conserving surgery

**Table 3:** Univariate and multivariate analyses of overall survival

Clinicopathological parameter	Hazard ratio	95% CI	*P*
Univariate analysis			
Age (≤ 35 versus 36–75 versus > 75)	≤ 35 versus 36–75: 4.48 ≤ 35 versus > 75:5.15	≤ 35 versus 36–75: 0.61–33.03 ≤ 35 versus > 75:0.32–82.36	0.165
Pathology IDC versus others	4.60	0.62–33.84	0.134
Grade III versus I–II	0.76	0.35–1.66	0.490
Ki67 > 30% versus ≤ 30%	1.02	0.46–2.26	0.957
cT2–T4 versus cT1	5.91	1.40–24.91	0.016
cN + versus cN0	3.82	1.32–11.04	0.013
Breast/ovarian cancer family history versus not	0.54	0.16–1.78	0.308
Family history of other cancers versus not	1.38	0.42–4.61	0.596
Second primary cancer versus not	0.84	0.11–6.20	0.866
Chemotherapy versus not	0.83	0.11–6.15	0.856
Radiotherapy versus not	2.26	0.86–5.96	0.098
HRD + versus HRD-	0.48	0.22–1.05	0.067
Multivariate analysis			
cT2–T4 versus cT1	4.41	1.02–18.99	0.047
cN + versus cN0	3.02	1.03–8.85	0.044
HRD + versus HRD-	0.45	0.20–0.99	0.049

*HRD* homologous recombination deficiency, *BCS* breast-conserving surgery

## Discussion

The present study analyzed the frequency of HRD and its association with carboplatin treatment response in early-stage TNBC patients. We found that HRD was identified in 53.3% of early TNBC patients, which was similar to previous reports [18, 27, 28]. Additionally, we also found that HRD status was significantly associated with high Ki67 levels in TNBC patients, similar to the results reported for breast cancer [5] and other solid tumors [29], which indicated high proliferation potential in HRD cancer cells. Moreover, we found that HRD positivity or a high HRD score was significantly associated with a higher pCR rate, especially in patients treated with carboplatin-containing neoadjuvant regimens. Furthermore, we found that HRD positivity was associated with favorable DMFS and OS, irrespective of carboplatin treatment.

The HRD score was calculated on the basis of loss of heterozygosity, telomeric allelic imbalance, and large-scale state transitions, which indicate the effects of the HRD pathway. HRD can be caused by several genetic and epigenetic changes, including *BRCA1/2* gene mutations, *BRCA*1 promoter methylation, HRR gene mutations, and epigenetic changes in HRR genes [30, 31]. However, the associations between non-*BRCA* HRR genes and HRD scores have not been well established. Our study analyzed the association of HRR gene mutations with HRD scores. The *BRCA1/2*-mutated group had a greater HRD score than the wild-type group, indicating the core role of the BRCA protein in maintaining the process of homologous recombination [18]. Interestingly, there was no significant difference in the HRD score between the non-*BRCA* HRR gene mutation group and the wild-type group, indicating that *BRCA1/2* are the predominant driver genes for constructing the HRD score. Our results showed that the genomic level of non-*BRCA* HRR genes, such as *PALB2* and *CHEK2*, did not greatly contribute to a high HRD score. These results indicated that HRD positivity may also be caused by epigenetic alterations or posttranslational modifications of non-*BRCA* HRR genes [32–34], rather than mutations of these genes, which needs further validation in future studies.

Notably, in the neoadjuvant cohort, patients with HRD-positive tumors had a greater pCR rate than did those with HRD-negative tumors, supporting the superior chemotherapy response and survival benefit in patients with HRD. In the GeparSixto trial, TNBC patients with HRD were independently related to a higher pCR rate after receiving neoadjuvant therapy [20]. Another pooled analysis of five phase II studies also showed that HRD was significantly associated with a higher pCR rate and RCB 0/I in TNBC patients [32]. Thus, HRD status could serve as a potential predictor of NAC efficacy in TNBC patients. Moreover, in patients who received the carboplatin-containing NAC regimen, HRD was significantly associated with a higher pCR rate. Our results were in line with those of the PrECOG 0105 study, which concluded that TNBC patients with higher HRDs were more likely to achieve pCR after neoadjuvant therapy [33]. Increasing evidence has demonstrated that HRD could predict both greater response and survival in favor of carboplatin over other common chemotherapy agents, including docetaxel and epirubicin [34]. There is a strong association between *BRCA1* mutation and basal-like cancer [35], which shares similar features with high degrees of chromosomal genomic instability [36], which might indicate the high platinum sensitivity we found in the HRD-positive carriers. However, in patients who received the noncarboplatin NAC regimen, the pCR rate tended to increase in the HRD-positive group, indicating that HRD status was not an ideal factor for predicting carboplatin treatment efficacy.

Regarding prognosis, we found that a high HRD score was associated with favorable disease outcomes in early TNBC patients, which was consistent with the findings of previous studies of other solid tumors, including ovarian cancer [37, 38], colorectal cancer [39], and pancreatic adenocarcinoma [40]. Possible mechanisms could be explained as follows: first, HRD-positive patients showed a better response to chemotherapy, including anthracycline/taxane-based and platinum-containing regimens. Since more than 97% of the enrolled TNBC patients received chemotherapy in the present study, patients with HRD would have a better chemotherapy response and prognosis. Second, patients with high HRDs were found to have greater immune cell infiltration [41, 42]. DNA damage can regulate the cGAS-STING pathway and recruit tumor-infiltrating lymphocytes, consequently activating antitumor immune responses and leading to favorable patient survival [40]. Interestingly, we found that HRD-positive carriers had significantly better DMFS and OS regardless of carboplatin usage. A meta-analysis encompassing more than 300 *BRCA*-mutated patients demonstrated that germline *BRCA* mutation carriers would receive no benefit from platinum usage [43], which could be explained by the fact that current standard adjuvant chemotherapy for TNBC patients already contains DNA-damaging agents, such as alkylants or anthracyclines, which may decrease the efficacy of carboplatin in *BRCA*-mutated patients.

Overall, the main findings of our research have several clinical and translational implications for TNBC treatment. Since there is no officially approved HRD test for breast cancer, our study enrolled a large number of early TNBC patients and supported that the original HRD test system is feasible for these patients. Potential limitations may exist in this study due to its retrospective design. First, our research is only a single-center study that continuously included TNBC patients who have undergone surgery and (neo)adjuvant therapy, and further multicenter studies are needed to confirm the major findings. Second, relatively small amount of patients with special histological type were included in the current analysis and the intrinsic subtype information was also lacked due to retrospective data, and further exploration is needed. Besides, chemotherapy backbone of patients received platinum agents were not consistent in the current study, and sample size was relatively small for further subgroup analysis. Furthermore, since there is no standard method for detecting HRD status in breast cancer patients, comparisons between this original HRD testing method and other detection methods were not performed, and future prospective studies are needed to integrate these methods to evaluate their clinical significance.

## Conclusion

High HRDs were related to high Ki67 levels and *BRCA* mutations in TNBC patients. HRD-positive TNBC patients treated with carboplatin had a higher pCR rate. Patients with HRD positivity had a better prognosis, irrespective of carboplatin treatment, warranting further evaluation.

## Supplementary Information

Below is the link to the electronic supplementary material.

Supplementary file1 (PPTX 11591 KB)

Supplementary file2 (DOCX 17 KB)

## Data Availability

The data analyzed in the current study are available from the corresponding authors on reasonable request.
