# Supplementary material for: Association between homologous recombination deficiency status and carboplatin treatment response in early triple-negative breast cancer
Source: Breast Cancer Res Treat. 2024 Jul 24;208(2):429–40. doi: 10.1007/s10549-024-07436-1 (PMC11457550; doi:10.1007/s10549-024-07436-1)
Supplement: Supplementary file 1 — Supplementary file1 (PPTX 11591 KB) [file 10549_2024_7436_MOESM1_ESM.pptx]

## Slide 1
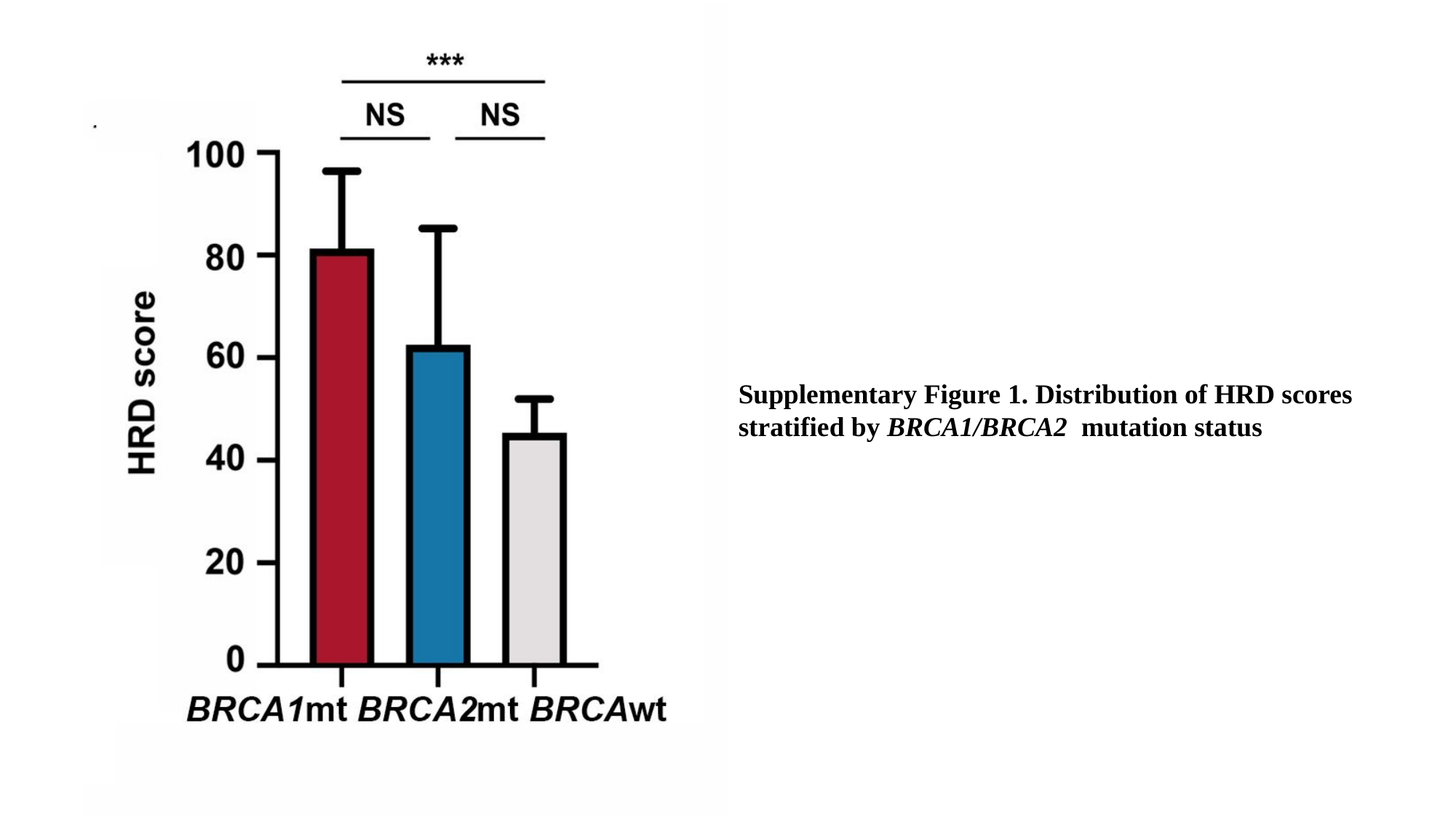

Supplementary Figure 1. Distribution of HRD scores stratified by BRCA1/BRCA2 mutation status

## Slide 2
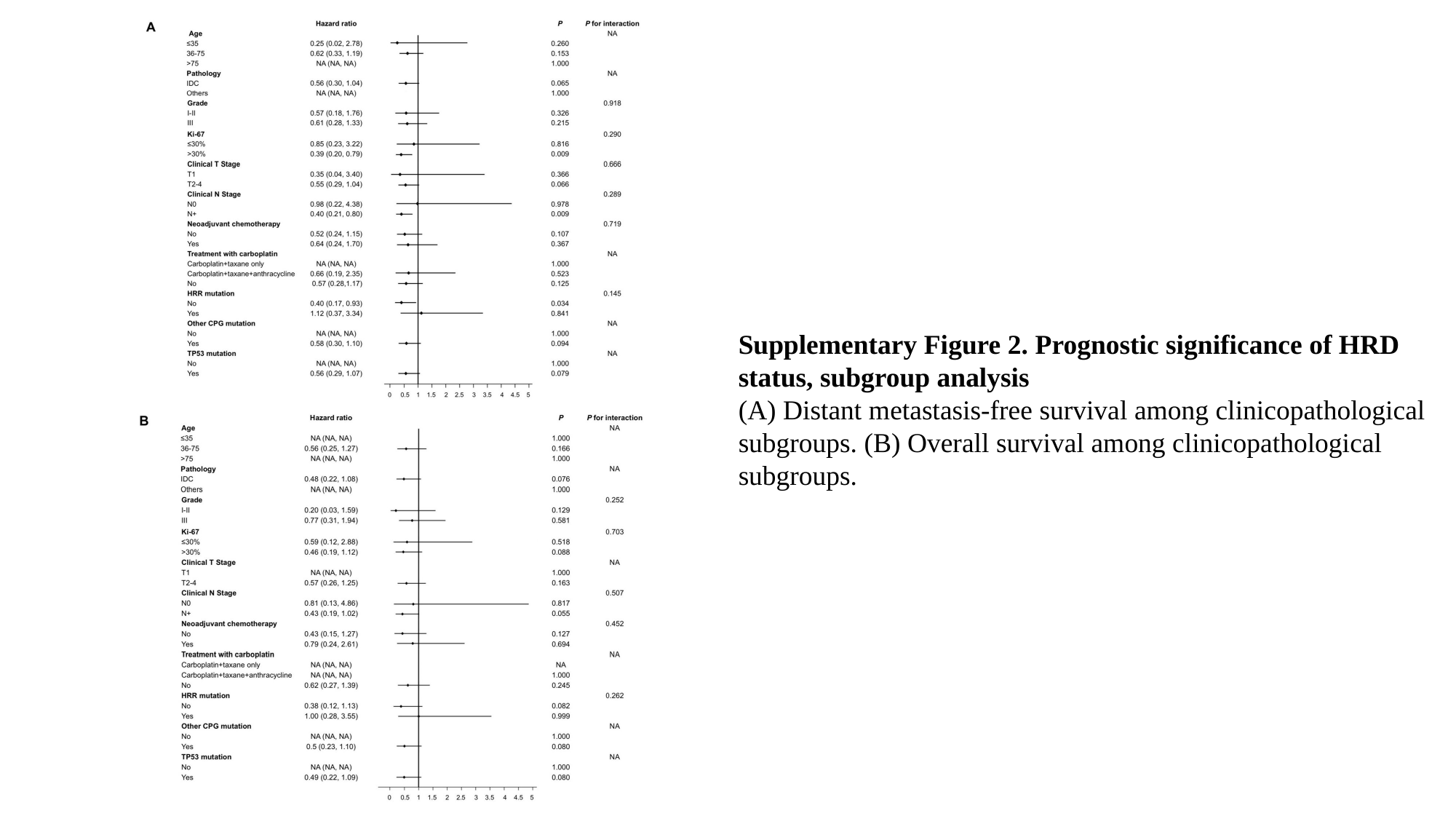

Supplementary Figure 2. Prognostic significance of HRD status, subgroup analysis
(A) Distant metastasis-free survival among clinicopathological subgroups. (B) Overall survival among clinicopathological subgroups.

## Slide 3
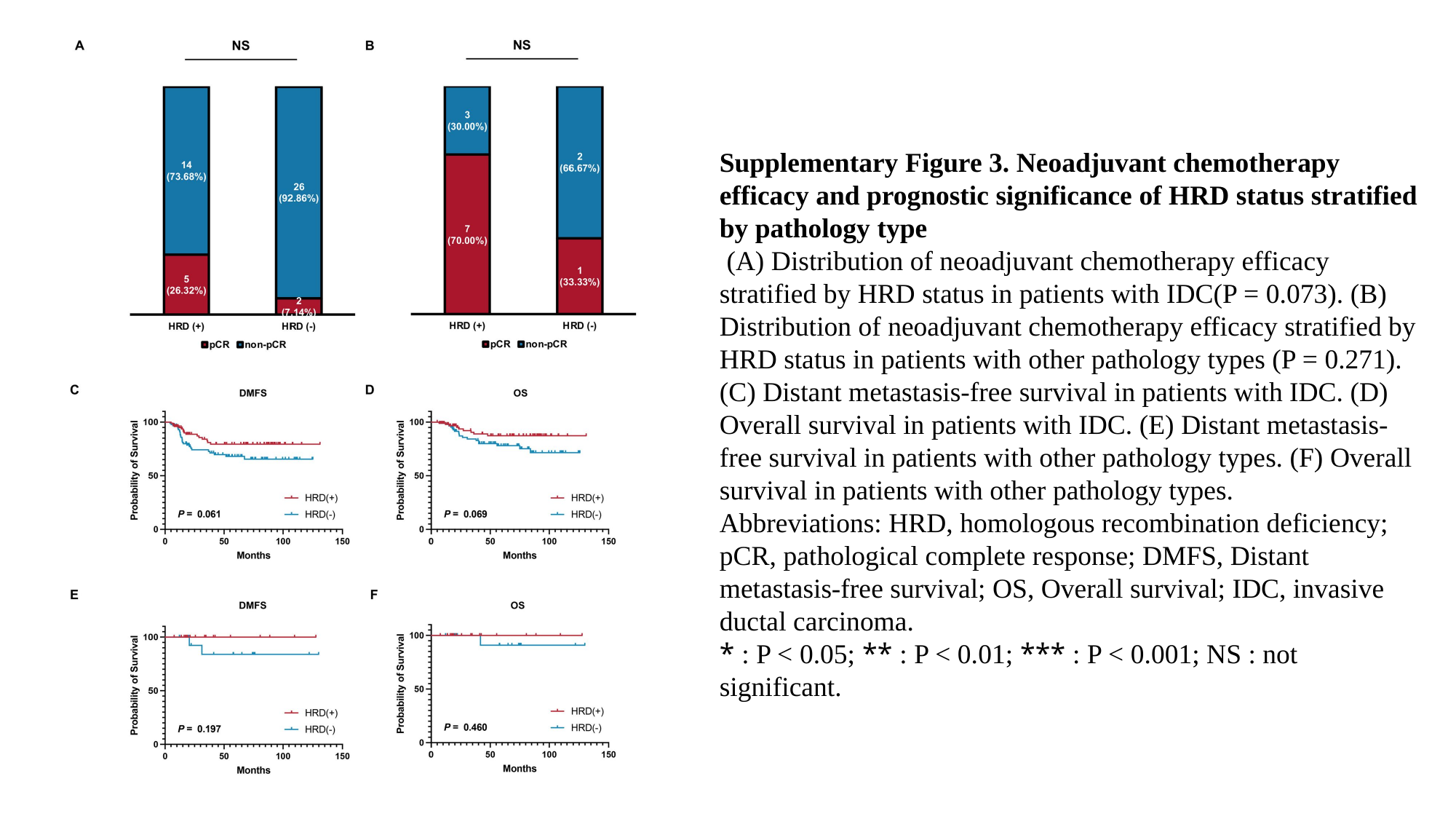

Supplementary Figure 3. Neoadjuvant chemotherapy efficacy and prognostic significance of HRD status stratified by pathology type
 (A) Distribution of neoadjuvant chemotherapy efficacy stratified by HRD status in patients with IDC(P = 0.073). (B) Distribution of neoadjuvant chemotherapy efficacy stratified by HRD status in patients with other pathology types (P = 0.271). (C) Distant metastasis-free survival in patients with IDC. (D) Overall survival in patients with IDC. (E) Distant metastasis-free survival in patients with other pathology types. (F) Overall survival in patients with other pathology types.
Abbreviations: HRD, homologous recombination deficiency; pCR, pathological complete response; DMFS, Distant metastasis-free survival; OS, Overall survival; IDC, invasive ductal carcinoma.
* : P < 0.05; ** : P < 0.01; *** : P < 0.001; NS : not significant.

## Slide 4
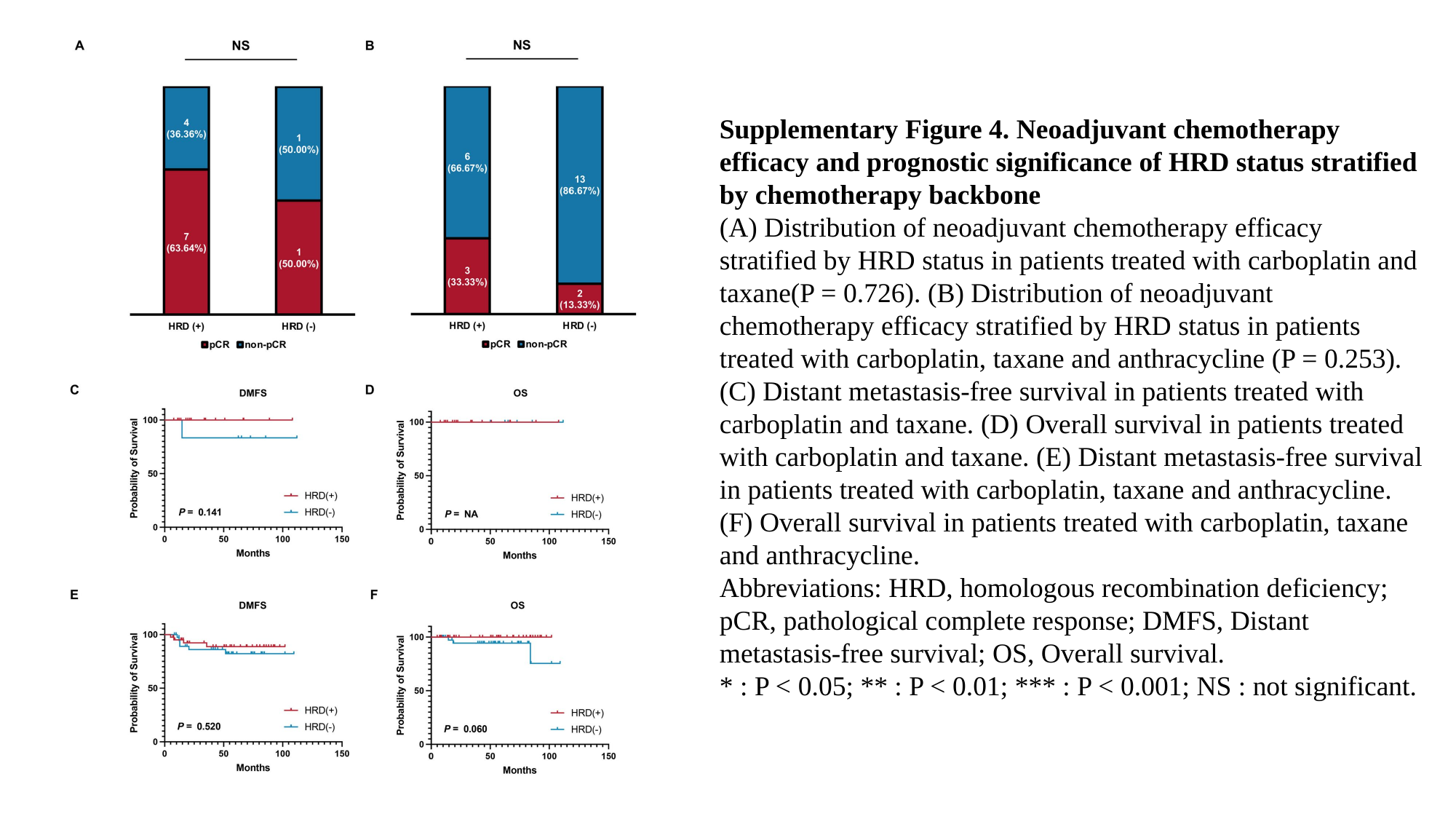

Supplementary Figure 4. Neoadjuvant chemotherapy efficacy and prognostic significance of HRD status stratified by chemotherapy backbone
(A) Distribution of neoadjuvant chemotherapy efficacy stratified by HRD status in patients treated with carboplatin and taxane(P = 0.726). (B) Distribution of neoadjuvant chemotherapy efficacy stratified by HRD status in patients treated with carboplatin, taxane and anthracycline (P = 0.253). (C) Distant metastasis-free survival in patients treated with carboplatin and taxane. (D) Overall survival in patients treated with carboplatin and taxane. (E) Distant metastasis-free survival in patients treated with carboplatin, taxane and anthracycline. (F) Overall survival in patients treated with carboplatin, taxane and anthracycline.
Abbreviations: HRD, homologous recombination deficiency; pCR, pathological complete response; DMFS, Distant metastasis-free survival; OS, Overall survival.
* : P < 0.05; ** : P < 0.01; *** : P < 0.001; NS : not significant.

## Slide 5
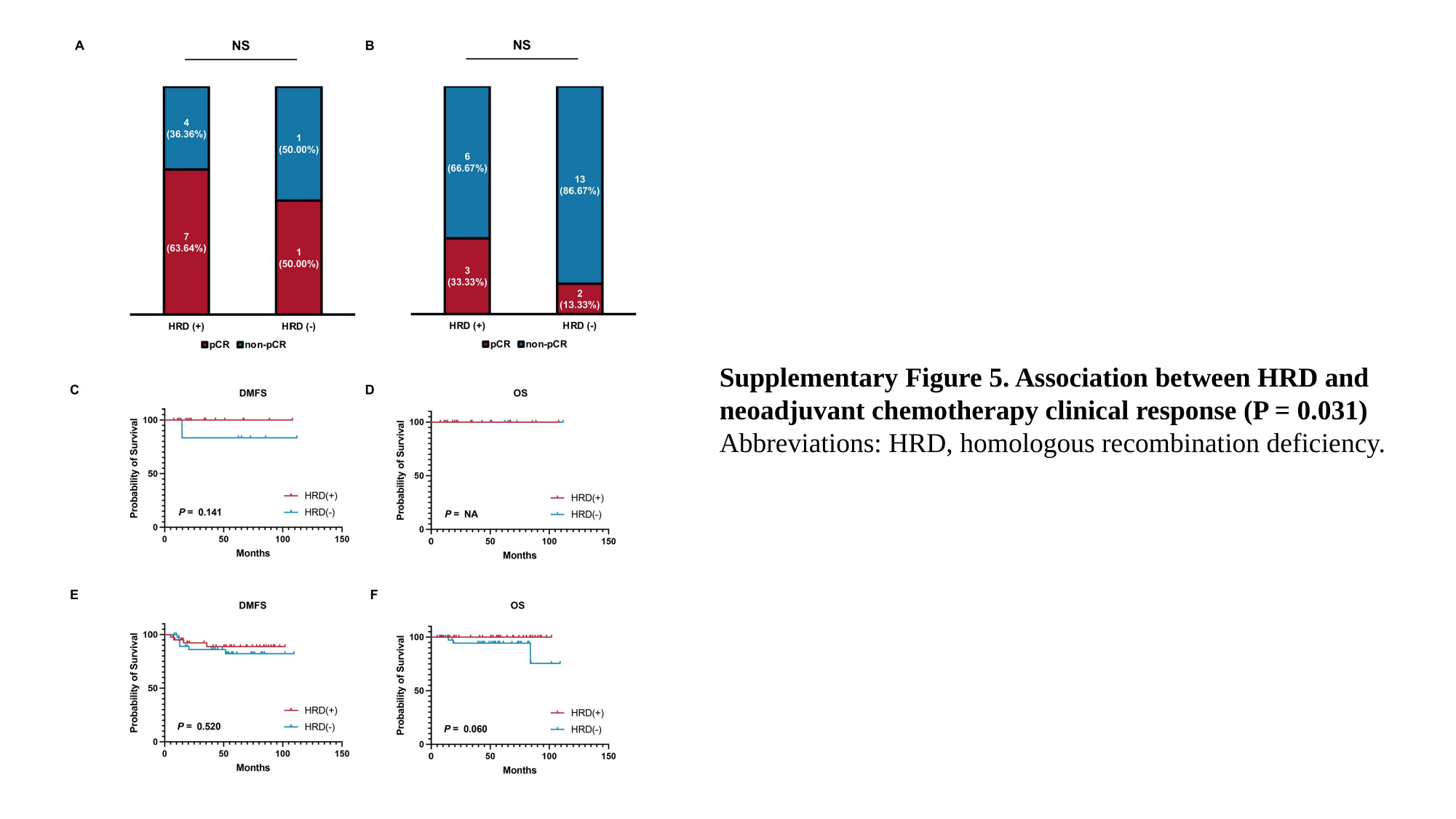

Supplementary Figure 5. Association between HRD and neoadjuvant chemotherapy clinical response (P = 0.031)
Abbreviations: HRD, homologous recombination deficiency.
