# Supplementary material for: Association between homologous recombination deficiency status and carboplatin treatment response in early triple-negative breast cancer
Source: Breast Cancer Res Treat. 2024 Jul 24;208(2):429–40. doi: 10.1007/s10549-024-07436-1 (PMC11457550; doi:10.1007/s10549-024-07436-1)
Supplement: Supplementary file 2 — Supplementary file2 (DOCX 17 KB) [file 10549_2024_7436_MOESM2_ESM.docx]

**Supplementary Table 1. Frequency of HRR and other cancer predisposition gene mutations**

| **Gene** | **Frequency** | **Mutation rate (%)** |
| --- | --- | --- |
| ***ATM*** | 9 | 4.00 |
| ***BARD1*** | 3 | 1.33 |
| ***BRCA1*** | 25 | 11.11 |
| ***BRCA2*** | 19 | 8.44 |
| ***BRIP1*** | 5 | 2.22 |
| ***CDH1*** | 5 | 2.22 |
| ***CDK12*** | 5 | 2.22 |
| ***CHEK1*** | 6 | 2.67 |
| ***CHEK2*** | 3 | 1.33 |
| ***FANCA*** | 4 | 1.78 |
| ***FANCL*** | 2 | 0.89 |
| ***HDAC2*** | 1 | 0.44 |
| ***PALB2*** | 4 | 1.78 |
| ***PPP2R2A*** | 1 | 0.44 |
| ***PTEN*** | 18 | 8.00 |
| ***RAD51B*** | 1 | 0.44 |
| ***RAD51C*** | 3 | 1.33 |
| ***RAD51D*** | 1 | 0.44 |
| ***RAD54L*** | 2 | 0.89 |
| ***TP53*** | 187 | 83.11 |

Abbreviations: HRR, homologous recombination repair
